# A 10-Week Program of Combined Hippotherapy and Scroth’s Exercises Improves Balance and Postural Asymmetries in Adolescence Idiopathic Scoliosis: A Randomized Controlled Study

**DOI:** 10.3390/children9010023

**Published:** 2021-12-30

**Authors:** Amr A. Abdel-aziem, Osama R. Abdelraouf, Shahesta A. Ghally, Haytham A. Dahlawi, Rafik E. Radwan

**Affiliations:** 1 Department of Physical Therapy, College of Applied Medical Sciences, Taif University, P.O. Box 11099, Taif 21944, Saudi Arabia; amralmaz@tu.edu.sa; 2 Department of Biomechanics, Faculty of Physical Therapy, Cairo University, Giza 12613, Egypt; rafik_radwan@hotmail.com; 3 Department of Musculoskeletal Disorders and Its Surgery, Faculty of Physical Therapy, October 6 University, Giza 12585, Egypt; Shahesta.Ahmed.PT@o6u.edu.eg; 4 Clinical Laboratory Sciences Department, College of Applied Medical Sciences, Taif University, P.O. Box 11099, Taif 21944, Saudi Arabia; haytham.d@tu.edu.sa

**Keywords:** balance, hippotherapy, posture, Schroth exercises, adolescent idiopathic scoliosis

## Abstract

Introduction: The most frequent type of spine abnormality throughout adolescence was adolescent idiopathic scoliosis (AIS). Hippotherapy improved posture, balance and gait of different musculoskeletal conditions. Therefore, this study aims to see how hippotherapy combined with Schroth exercises affected postural asymmetry and dynamic balance in AIS compared to traditional physiotherapy (Schroth exercises) alone. Materials and methods: In this randomized controlled trial, fifty-two patients with AIS (10–18 years, 37 girls and 15 boys) participated. They were arbitrarily allocated into two groups: experimental (19 female/8 male; aged 14.74 ± 1.79 years; Cobb angle 18.59 ± 2.66 degrees) and control (18 female/7 male; aged 15.04 ± 1.81 years; Cobb angle 19.32 ± 2.69 degrees) groups. Both groups received Schroth exercises for 10 weeks, three days/week. The experimental group additionally received hippotherapy training. Pre-treatment and post-treatment assessment for the scoliotic, kyphotic angle, pelvic obliquity, pelvic torsion and vertical spinal rotation and the anteroposterior, mediolateral and overall stability indices were assessed using the formetric system 4D and Biodex Balance System, respectively. Results: After intervention, both groups illustrated significant improvements in all examined variables (*p* < 0.05). The experimental group illustrated significant improvements in scoliotic angle, kyphotic angle, pelvic obliquity, pelvic torsion and vertical spinal rotation and the stability indices compared to the control group (*p* < 0.05). Conclusion: In adolescence idiopathic scoliosis, hippotherapy training combined with Schroth exercises improves posture asymmetry and balancing ability more effectively than Schroth exercises alone.

## 1. Introduction

Adolescent idiopathic scoliosis (AIS) is a disease characterized by three-dimensional spinal malformation marked by deformation in the sagittal (thoracic lordosis), frontal (lateral curvature) and transverse planes (vertebral rotation). It is a classic orthopedic problem, which has crippling disorders affecting the previously healthy young children, and its treatment might be difficult [1,2,3]. The likelihood of spinal deformity worsening is higher the younger the kid is; also, pubertal development raises the possibility of spinal malformation progression [4]. Moreover, it causes an uneven load distribution in the vertebral body, which contributes to the disease’s progression [5]. At a certain stage, the onset and progression of idiopathic scoliosis is a multifaceted disorder, with genetics and the mechanics of the upright position of the spine playing a key responsibility [1,2]. The prevalence of AIS is 2.3% (female, 3.1%; male, 1.5%) in Turkey [6]. Moreover, in Sao Paulo state cities, its prevalence is analogous to the previous literature (female, 2.2 percent; male, 0.5 percent), which was higher among females, especially through adolescence (13–14 years of age) [7].

The postural balance might be affected by the imbalance of upper body postural alignment that occurred as a result of scoliosis [8,9]. However, the current literature on balance affection in patients with idiopathic scoliosis is not consistent. According to several studies, when compared to healthy people, those patients do not have postural balance disturbance [8,9,10,11,12]. Multiple investigations, however, have revealed that AIS has an effect on postural balance [10,13,14,15,16,17,18,19,20,21]. This inconsistency in results of the studies could be related to the disparity in curve types (single or double), curve placement (thoracic and lumbar) and embraced Cobb angles; these parameters have been found to affect the postural stability. Single lumbar curves, for example [22], and greater Cobb angle [23] are associated with poor postural balance.

Hippotherapy is a special form of physiotherapy technique, a specially modified form of horse riding designed for the management of stability and functional disorders [24]. The theory of hippotherapy is that a horse directs the riders through a repeating periodic movement pattern (similar to human walking) [25]. The patient is a passive part of hippotherapy, and their body movement responds to the horse’s movement [26]. According to Stergiou et al. [27], who concluded that for patients with balance or gait disturbances and even psychomotor impairments, hippotherapy is a feasible and alternative intervention.

Many studies reported that hippotherapy has been shown to recover walking abilities in multiple sclerosis subjects [28], balance in elderly subjects [29], postural balance in normal adults [30] and symmetry of muscle activation in cerebral palsy children [31]. Moreover, it improves the trunk alignment of patients with functional scoliosis, which is created by pelvis tilting and lumbar spine rotation [32]. Riding a horse can cause movement patterns in the human pelvis that are quite similar to those seen when walking normally [33].

However, there is limited literature data available concerning the effects of hippotherapy on balance and postural symmetry in those suffering from idiopathic scoliosis. Therefore, the purpose of this research work was to evaluate how hippotherapy combined with Schroth exercises affected dynamic balance and postural asymmetry in adolescents with idiopathic scoliosis compared to standard physiotherapy (Schroth exercises) alone.

## 2. Materials and Methods

### 2.1. Participants

This randomized experiment included 52 patients (15 males and 37 females) with AIS between the ages of 10 and 18 years old; they were diagnosed at puberty age by pediatric specialists, having spinal curves of all types, with Cobb angles ranging from 10 to 25 degrees. Those with a considerable history of horseback riding, other treatments that might cause scoliosis, chronic conditions necessitating drug use and the existence of any neurologic, muscular, or rheumatic diseases were all excluded.

In this research design, the participants were accidentally allocated into two groups; the experimental group received hippotherapy in combination with Schroth exercises, while the control group received only Schroth exercises. Allocation concealment was accomplished by placing the assignment in opaque, sealed envelopes that are consecutively numbered. The envelopes were opened in front of the participants by an outside independent person. G*Power 3.1 was used to calculate the sample size with an alpha of 0.05, a power (90%) and an effect size (0.5). A total of 44 patients were needed for the study.

**Figure 1:**
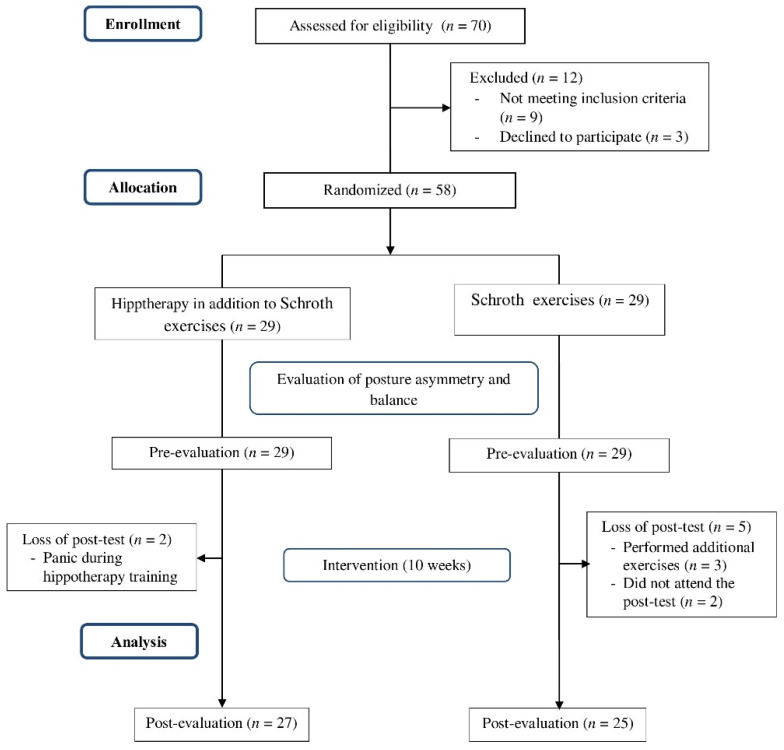
Flowchart of the study processes.

All the referred candidates attending the Biomechanics Lab to be screened for the inclusion criteria. The participants that met the criteria were randomly assigned into the experimental and control groups. Then, an independent blinded investigator assessed the baseline measurement of all outcomes and a 2-week schedule for Schroth home exercise training. After ten weeks of intervention, the participants attended the lab one more time for the post-intervention measurements of postural asymmetry and balance. Figure 1 depicts the subject’s flow through the various stages of the study.

Data, including age, sex, height and weight, were recorded and are shown in Table 1. Each participant signed a written informed consent form. The Batterjee Medical College Ethical Committee accepted the trial protocol (RES-2021- 0034), date of approval 2 March 2021, and ClinicalTrials.gov PRS registered it (NCT04885023). Helsinki Declaration’s principles were considered during its conduction.

### 2.2. Assessment Procedure

**Figure 2:**
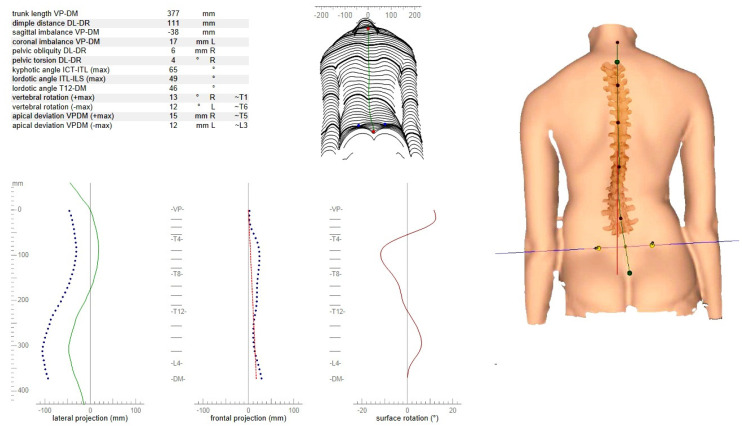
Raster-stereographic assessment of the spine and pelvis.

Raster-stereographic (Formetric) measurements: The Formetric 4D system discovered anatomical bony landmarks automatically, and the examiner did not change any of the landmark positions selected by the machine. When compared to radiology, the raste-stereographic system has a high level of validity, with outstanding intra and inter-observer reliability during the evaluation of AIS patients. Furthermore, instead of repeat radiographs, it is noninvasive for monitoring the progression of AIS in growing patients [34]. After calibration of the stereographic camera/projector, the stereographic camera/projector’s height could be adjusted to fit the subject’s height. After telling the participant to assume a relaxed standing posture for sixty seconds, the frames were captured (Figure 2). Each individual had three measures taken in a row, and the average was determined [35].

Balance evaluation: The postural stability test (PST) can be used to calculate the overall stability index (OSI), anterior-posterior stability index (APSI), mediolateral stability index (MLSI) and falling risk (FRT). These indices were evaluated using The Biodex Balance System (BBS, Biodex Inc., Shirley, NY, USA). The high results of these tests suggest a loss of balance and an increased danger of falling [36,37].

The tests were explained to each patient, and the instructions were to be followed. The patients were evaluated in three trials over the course of the study. Each trial lasted 20 s and was spaced by at least 10 s. The three tests’ average score was recorded. Before the test, the participants were given 3 min of BBS training to familiarize themselves with the equipment [38].

### 2.3. Intervention

**Figure 3:**
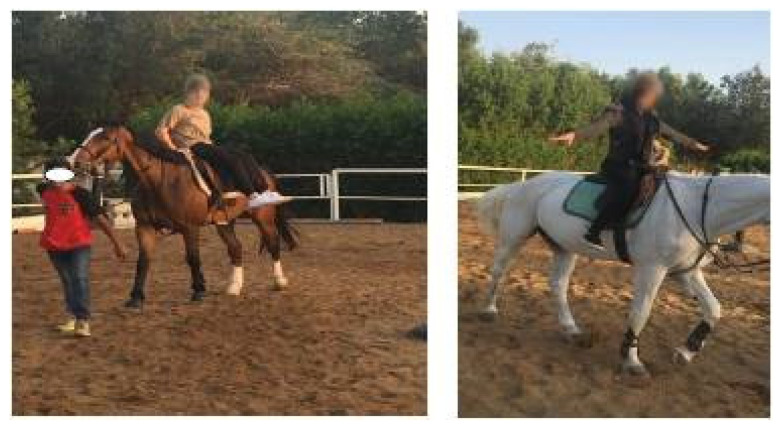
Horse-riding exercise training.

At two local therapeutic riding centers, interventions were carried out by a licensed physical therapist with hippotherapy experience. Over the course of ten weeks, the participants received 15 sessions, broken into two parts [39]. Once a week, In the first phase, they completed 30 min of walking and seated trot training without using a stirrup iron. For the remainder of the treatment program, the frequency was increased to twice a week. Two familiarization sessions were held prior to the commencement of the intervention to clarify safety standards and establish a riding connection with the horse. During each hippotherapy session, the participants wore protective helmets and mounted a moving horse at a walk or trot, to execute a variety of postures, such as forward astride, side sitting and backward astride, occasionally with changes between positions and frequently, even as the horse was moving under the physical therapist’s instructions, a leader yanked the horse reins in front of the horse to direct the gait velocity of horse and orient it in different directions (Figure 3).

Schroth exercises are asymmetric workouts with rotating breathing strategies for three-dimensional correction that are patient-specific; they were completed by both groups. For the first two weeks, the participants were trained in their home fitness routine over five 1-h individual sessions [40]. This was followed by weekly one-hour appointments, as well as daily home workouts. Modifications of the posture were aided by rotational breathing exercises focusing on the thorax concave side. During the therapy sessions, the postural correction principles of Schroth were used, which consisted of axial prolongation, deflexion, de-rotation, facilitation and stabilization [41].

### 2.4. Statistical Analysis

The data were analyzed using the Statistical Package for Social Sciences (SPSS) (Version 20.0, Armonk, NY, IBM Corp.). Before the final statistical analysis, the Shapiro–Wilk and Levene’s tests confirm the normal distribution and homogeneity of the data (*p* > 0.05). Therefore, the parametric data analysis was conducted. A Chi-square test was used to look into the noteworthy difference in gender, and an independent *t*-test was used to look into the noteworthy differences in age, sex, weight and height between both groups. The discrepancies between spinal Formetric measurements and stability indices in both groups were investigated using a two-way analysis of variance. The significance level of a *p*-value less than 0.05 was considered statistically significant using 95 percent confidence intervals.

## 3. Results

Table 1 demonstrated no noteworthy difference in age, height, weight or BMI between both groups (*p* > 0.05). In general, the between-subjects effect statistical analysis revealed no significant difference (groups; F = 1.04, *p* = 0.426); however, the within-subjects effect and interaction effect showed significant differences (time; F = 2.27, *p* = 0.001, time*group; F = 46.27, *p* = 0.001, respectively). The source of significance for groups interaction; experimental vs. control and time; pre-evaluation vs. post-evaluation were then determined using multiple pairwise comparison tests.

Table 2 illustrated that the pre-test values of scoliotic, kyphotic angle, pelvic obliquity, pelvic torsion and vertical spinal rotation of both groups were not statistically different (*p* > 0.05). The pre values of both groups were significantly greater than their post values (*p* < 0.05). Simultaneously, the experimental group’s post values were significantly lower than those of the control group (*p* < 0.05).

The pre values of the overall, anteroposterior and mediolateral stability indices of both groups were not substantially different (*p* > 0.05). Both groups’ pre values were substantially greater than their post values (*p* < 0.05). At the same time, the experimental group’s post values were significantly lower than those of the control group (*p* < 0.05). This is explained in Table 3.

## 4. Discussion

The results showed that both groups improved in all spinal measurements and dynamic postural stability indices. However, the experimental group made more progress than the control group. The experimental group improvement could be caused by hippotherapy’s development of neuromuscular coordination via the walking horse’s impulses carried as inputs to the rider’s central nervous system. It has been suggested that the horse’s repeating movement pattern mimics the human ambulating gait [42]. Similarly, the horse’s pelvis has a similar 3D motion as the human pelvis during walking, particularly its vertical displacement [43].

Moreover, Funakoshi et al. [44] reported that horse mounting increased the dynamic trunk alignment of healthy subjects. The understandable underlying mechanism can be expounded in the following: the horse motion created a rotating motion along the longitudinal axis, creating lateral pelvic tilting of the rider, indicating bending and rotation of the spine, generating longitudinal motion of the neck. This process was not observed in exercises utilizing a horse-riding simulator, which could explain why real horses were used in this investigation.

Previous research has shown that in children with cerebral palsy, horse-riding activities improved weight shifting and postural control, as well as greater midline head control [24,45]. Furthermore, It was more successful than Kendall’s exercise in improving forward head posture deformity [46], and eight minutes of hippotherapy increased the symmetry of trunk muscular activation during sitting, standing and walking [31]. Moreover, it has been found that hippotherapy aids in the reduction of pain and inability in people suffering from back pain [47] and balances the trunk flexor/extensor ratio [48]. The Stergiou et al. [27] review study concluded that hippotherapy was beneficial for improving the gross and fine motor function, balance issues, walking capability, hypertonicity and body equilibrium. Furthermore, it enhances cognition state, emotional condition and social well-being.

The experimental group improved their balance more than the control group, which was consistent with reports by Araujo et al. [49] and Homnick et al. [50], who found horseback riding has a favorable effect on static balance and locomotion in the elderly when compared to conventional walking. It also increases motor coordination, muscle strength and other fitness-related factors [51]. This could be explained by the effect of hippotherapy on the symmetrical activation of muscles of the abdominal wall (rectus abdominis, internal oblique and transverse abdominis), muscles of the back (erector spinae, iliocostalis and multifidus), and muscles of the thigh (rectus femoris, tensor fascia latae, gluteus medius and adductor) [52,53]. They are the trunk core muscles that are important for enhancing postural control in young adults [54].

The trunk muscles, particularly the rectus abdominis, have been demonstrated to coordinate their activation to the horse’s movement [55], which coordinates body segments for posture [30]. The internal oblique helps to control posture by increasing intraabdominal pressure, which is aided by the rectus abdominis and transverse abdominis [56]. Terada et al. [55] reported that the horse riding elicited a greater adductor muscle activity, and its rectus abdominis activation uses the hip flexion to control the posture [30], which explains the greater improvement of pelvis posture of the experimental group. Furthermore, horseback riding alters muscle use and joint motions, perhaps leading to increased adductor muscle activity [57]. It is possible that when horseback riding, the hip adductor muscle was heavily exercised. The activity of the trunk muscles was regulated consequently of acclimatization to the horse movements.

The control group improvement in posture and body balance could be assigned to Schroth exercises’ effect, which is well-known as patient-specific asymmetric exercises used for three-dimensional posture correction through rotating breathing techniques. These exercises involve asymmetrical standing postural exercises that were designed to help postural control by restoring body balance and mobility [41]. Schreiber et al. [58] found that the Schroth exercises have a superior effect on correction of the curve’s severity compared to traditional treatment in patients with AIS. Moreover, Kuru et al. [59] stated that Schroth’s exercises were found to be more successful than home exercise programs in reducing Cobb and rotation angles, as well as trunk asymmetry, under the condition that it was performed in a clinic under the supervision of a physiotherapist. In addition, Schreiber et al. [60] found that Schroth exercises, when used in conjunction with normal therapy, were found to improve the severity of the spinal curve misalignment (Cobb angle), vertebral rotation of the vertebrae, enhance the back muscles endurance and body posture of adolescents suffering from idiopathic scoliosis.

Despite the bracing treatment’s long-term success, several authors have pointed out potential downsides, such as a temporary reduction in pulmonary functions and restrictions in the maximum capacity of exercises, particularly in girls [61,62]. However, bracing was advised for the treatment of AIS, especially for individuals having a Cobb angle greater than 20°, or most commonly between 20 and 40° [63,64]. Since our participants had mild AIS, their Cobb angles ranged between 10 and 25 degrees, the current study did not assess the efficacy of bracing when added to the treatment program of both groups. In addition, there are no respiratory concerns in those suffering from moderate scoliosis and a Cobb angle of 26 degrees [65], which was coincident with the current study.

This research work was limited to the following: the absence of long-term follow-up for participants in both groups. Therefore, similar studies should be conducted to monitor the study’s outcomes after the trial ends for a period of three to six months to determine the long-term effects of both interventions. Muscle strength, gait characteristics and energy expenditure were not measured as secondary outcomes in this study. Therefore, if these variables are included in future studies, they will be beneficial to adolescents with idiopathic scoliosis. In the future, electromyography studies will be required to document the effect of hippotherapy combined with Schroth exercises on the trunk muscles’ activities in people with AIS. A comparative study is also recommended to compare the impact of riding a live horse to a simulator on the measured outcomes, as riding a simulator is less expensive and may be more familiar to certain participants. Finally, more study is needed to establish the impact of hippotherapy training on psychological indicators, such as quality of life in participants.

## 5. Conclusions

In teenagers with idiopathic scoliosis, combining hippotherapy with Schroth exercises improves postural asymmetry and balancing ability more effectively than Schroth exercises alone. As a result, while developing an AIS rehabilitation program, this combination should be taken into account.

## Figures and Tables

**Table 1:** Participants’ demographic characteristics.

Groups	Experimental Group, *n* = 27	Control Group B, *n* = 25	*p*-Value
Age, years	14.74 ± 1.79	15.04 ± 1.81	0.552 ^a^
Height, cm	157.22 ± 6.88	158.00 ± 7.27	0.693 ^a^
Weight, kg	48.99 ± 5.15	50.54 ± 5.28	0.289 ^a^
Body mass index, kg/m^2^	19.77 ± 1.02	20.21 ± 1.05	0.133 ^a^
Cobb angle, degree	18.59 ± 2.66	19.32 ± 2.69	0.332 ^a^
Gender, male/female	8/19	7/18	0.897 ^b^

*p*-value > 0.05 indicates non-significant difference, ^a^ refers to independent *t*-test, ^b^ refers to Chi-square test, the data are shown as mean ± standard deviation.

**Table 2:** Descriptive statistics of the formetric measurements before and after intervention.

Variables		Experimental Group, *n* = 27	Control Group, *n* = 25	*p*-Value
Scoliotic angle, degrees	Pre	24.09 ± 5.50	25.06 ± 5.24	0.520
	Post	18.41 ± 5.42	22.32 ± 4.73	0.008 *
	*p*-value	0.001 *	0.001 *	
Kyphotic angle, degrees	Pre	49.26 ± 6.19	50.32 ± 5.76	0.526
	Post	44.26 ± 5.47	48.00 ± 5.45	0.017 *
	*p*-value	0.001 *	0.001 *	
Pelvic obliquity, degrees	Pre	4.91 ± 1.46	4.99 ± 1.38	0.838
	Post	2.37 ± 1.05	3.08 ± 0.90	0.012 *
	*p*-value	0.001 *	0.001 *	
Pelvic torsion, degrees	Pre	1.92 ± 0.82	1.95 ± 0.81	0.897
	Post	1.07 ± 0.55	1.51 ± 0.70	0.013 *
	*p*-value	0.001 *	0.001 *	
Vertical rotation (RMS), degrees	Pre	4.09 ± 1.49	4.30 ± 1.35	0.602
	Post	2.51 ± 1.14	3.34 ± 1.12	0.011 *
	*p*-value	0.001 *	0.001 *	

* *p*-value < 0.05 indicates a significant difference, RMS stands for root mean square, and the data are shown as mean ± standard deviation.

**Table 3:** Descriptive statistics of dynamic postural stability indices before and after intervention.

Variables		Experimental Group, *n* = 27	Control Group, *n* = 25	*p*-Value
Overall stability index	Pre	0.39 ± 0.11	0.41 ± 0.12	0.632
	Post	0.32 ± 0.10	0.39 ± 0.11	0.024 *
	*p*-value	0.001 *	0.001 *	
Anteroposterior stability index	Pre	0.28 ± 0.11	0.29 ± 0.12	0.746
	Post	0.21 ± 0.10	0.28 ± 0.11	0.033*
	*p*-value	0.001 *	0.001 *	
Mediolateral stability index	Pre	0.24 ± 0.11	0.25 ± 0.12	0.751
	Post	0.18 ± 0.09	0.24 ± 0.11	0.041 *
	*p*-value	0.001 *	0.012 *	

* *p*-value < 0.05 indicates a significant difference, RMS stands for root mean square, and the data are shown as mean ± standard deviation.

## Data Availability

Data available on request.
